# Usmani–Riazuddin Syndrome: Functional Characterization of a Novel c.196G>A Variant in the *AP1G1* Gene and Phenotypic Insights Using Zebrafish as a Vertebrate Model

**DOI:** 10.3390/ijms262110590

**Published:** 2025-10-30

**Authors:** Valentina Imperatore, Alessandra Mirarchi, Emanuele Agolini, Andrea Astolfi, Maria Letizia Barreca, Antonio Novelli, Elisa Vinciarelli, Sara Ferretti, Daniela Zizioli, Giuseppe Borsani, Cataldo Arcuri, Paolo Prontera

**Affiliations:** 1 Department of Medicine and Surgery, University of Perugia, 06123 Perugia, Italy; valentina.imperatore@unipg.it (V.I.); alessandra.mirarchi@unipg.it (A.M.); cataldo.arcuri@unipg.it (C.A.); 2 Laboratory of Medical Genetics, Translational Cytogenomics Research Unit, Bambino Gesù Children’s Hospital, IRCCS, 00168 Rome, Italy; emanuele.agolini@opbg.net (E.A.); antonio.novelli@opbg.net (A.N.); 3 Department of Pharmaceutical Sciences, University of Perugia, 06123 Perugia, Italy; andrea.astolfi@unipg.it (A.A.); maria.barreca@unipg.it (M.L.B.); 4 Medical Genetics Unit, Azienda Ospedaliera S. Maria Della Misericordia of Perugia, 06129 Perugia, Italy; elisa.vinciarelli@virgilio.it; 5 Department of Molecular and Translational Medicine, University of Brescia, 25123 Brescia, Italy; s.ferretti002@studenti.unibs.it (S.F.); daniela.zizioli@unibs.it (D.Z.); giuseppe.borsani@unibs.it (G.B.)

**Keywords:** brain disorder, Usmani–Riazzudin syndrome, USRIS, intellectual disability, neurodevelopmental delay, AP1G1, whole-exome sequencing, zebrafish, central nervous system, locomotor behavior

## Abstract

Adaptor Protein-1 (AP-1) is a heterotetrameric essential for intracellular vesicular trafficking and polarized localization of somato-dendritic proteins in neurons. Variants in the *AP1G1* gene, encoding the gamma-1 subunit of adaptor-related protein complex 1 (AP1γ1), have recently been associated with Usmani–Riazuddin syndrome (USRISD, MIM#619467), a very rare human genetic disorder characterized by intellectual disability (ID), speech and neurodevelopmental delays. Here we report a novel variant (c.196G>A; p.Gly66Arg) identified by exome sequencing analysis in a young girl showing overlapping clinical features with USRIS, such as motor and speech delay, intellectual disability and abnormal aggressive behavior. In silico analysis of the missense de novo variant suggested an alteration in AP1G1 protein folding. Patient’s fibroblasts have been studied with immunofluorescence techniques to analyze the intracellular distribution of AP-1. Zebrafish are widely regarded as an excellent vertebrate model for studying human disease pathogenesis, given their transparent embryonic development, ease of breeding, high genetic similarity to humans, and straightforward genetic manipulation. Leveraging these advantages, we investigated the phenotype, locomotor behavior, and CNS development in zebrafish embryos following the microinjection of human wild-type and mutated *AP1G1* mRNAs at the one-cell stage. Knockout (KO) of the *AP1G1* gene in zebrafish led to death at the gastrula stage. Lethality in the KO *AP1G1* fish model was significantly rescued by injection of the human wild-type *AP1G1* mRNA, but not by transcripts encoded by the Gly66Arg missense allele. The phenotype was also not rescued when *ap1g1−/−* zebrafish embryos were co-injected with both human wild-type and mutated mRNAs, supporting the dominant-negative effect of the new variant. In this study, we defined the effects of a new *AP1G1* variant in cellular and animal models of Usmani–Riazzudin syndrome for future therapeutic approaches.

## 1. Introduction

Usmani–Riazzudin syndrome (USRIS, OMIM 619467) (https://www.omim.org/entry/619467, accessed on 27 October 2025) is a rare autosomal neurodevelopmental disorder, described for the first time only a few years ago [1]. Its dominant and recessive forms have been reported in a cohort of patients showing overlapping clinical features. In 2021, Usmani et al. identified and studied nine patients with de novo dominant missense, splice site, and frameshift variants and two patients with biallelic, recessive, and missense variants.

The clinical phenotype is characterized by common signs such as intellectual disability, speech delay, developmental delay, hypotonia, and behavioral problems (mainly aggressive behavior), variably associated with congenital anomalies, epilepsy, spasticity, autism, bone abnormalities, vertebral and limb defects, and variable facial features (eyes and ear shape anomalies). Recently, an additional de novo variant was identified in a patient with multisystemic involvement and specific facial features, including synophrys, convergents quint, hypotelorism, upslanted palpebral fissures, broad nasal tip, thick upper and lower lip vermilion, and abnormal teeth [2], thus defining a more characteristic facial dysmorphism of the disease. The *AP1G1* gene is located on human chr16q22.2 and encodes the gamma-1 (γ) adaptor protein subunit of the AP-1 complex (AP1γ1, a protein of 822 amino acids). AP1γ1 is predicted to have four transmembrane domains, followed by a carboxyl-terminal alpha-adaptin C2 domain, which binds to clathrin and other receptors in coated vesicles [3,4]. Ubiquitous adaptor proteins (APs) are required for assembly, cargo sorting, and vesicular transport among endosomes, lysosomes, trans-Golgi networks (TGNs), and the plasma membrane [5,6,7,8]. It has been shown that AP-1 complexes not only are involved in these cellular functions in eukaryotic cells [9,10], but also mediate basolateral sorting in polarized epithelial cells [11,12,13,14], and transmembrane protein transport to somato-dendritic domains in neurons [15,16,17]. Loss of AP-1 complex subunits in murine models has been previously associated with death or severe growth deficits [11,16,18,19,20]. Notably, the AP1 complex plays a critical role in neural function, as evidenced by the fact that its dysregulation is a common hallmark of several neurological diseases [21].

Rare diseases are characterized by a low prevalence, which often means that patients with such diseases are undiagnosed and do not have effective treatment options. Neurodevelopmental and neurological disorders comprise approximately 40% of rare diseases [22], and in recent years, attempts have been made to identify the genes and mechanisms linked to these pathologies. This highlights the importance of using an animal model that can recapitulate the phenotype of rare diseases and, therefore, provides an excellent tool for elucidating the role of the genes involved, revealing mechanisms, and assessing therapeutic methods for rare diseases. Zebrafish (*Danio rerio*), a vertebrate with transparent embryos that develop externally, has emerged as an excellent model for studying different biological processes involved in central nervous system pathologies owing to advancements in CRISPR/Cas9 genome editing and transgenesis technologies [23]. Recently, our research group [24] generated a zebrafish *ap1g1* gene knockout using CRISPR/Cas9 genome editing to better understand the functional role of *AP1G1* in adaptinopathies. Heterozygous females and males showed morphological alterations in the brain, gonads, and intestinal epithelium. Analysis of the mRNA expression profiles of marker proteins, along with altered tissue morphology, revealed dysregulated cadherin-mediated cell adhesion. These findings highlight the utility of the zebrafish model for studying the molecular mechanisms of adaptinopathies and developing potential treatment strategies.

This study describes and characterizes another new missense variant (c.196G>A, p.Gly66Arg) in *AP1G1* in a girl with intellectual disability and abnormal behavior, thus expanding the case history. The phenotype of the zebrafish model expressing the human mutated allele is consistent with the clinical features of the patient, supporting a possible dominant-negative effect of the new mutation.

## 2. Results

### 2.1. Genetic Profile

**Figure 1:**
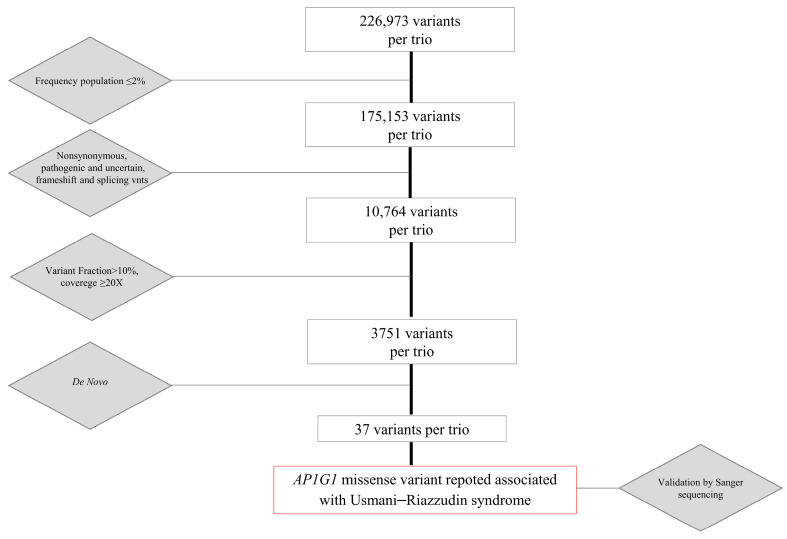
Flowchart showing variants prioritization process, that led to the identification of the *AP1G1* missense variant c.196G>A, p.Gly66Arg.

We conducted *exome sequencing* on the proband and her parents using DNA extracted from peripheral blood, as previously described [25]. Exome sequencing yielded approximately 226,973 genetic variants for the trio for further investigation. We filtered for variants with allele frequencies in the population <2% or not reported. Among them, we selected only variants classified as likely pathogenic (class IV), pathogenic (class V), or uncertain (class III-uncertain significance variant, VUS) according to the American College of Medical Genetics and Genomics (ACMG) classification criteria [26] in the available databases such as ClinVar (https://www.ncbi.nlm.nih.gov/clinvar/, accessed on 27 October 2025), ClinVar Miner (https://clinvarminer.genetics.utah.edu/, accessed on 27 October 2025), Leiden Open Variation Database (LOVD, https://www.lovd.nl/, accessed on 27 October 2025), or Human Gene Mutation Database (HGMD, https://www.hgmd.cf.ac.uk/ac/index.php, accessed on 27 October 2025), or not reported. We then filtered for variants that were either loss-of-function (frameshift, splice, stopgain, or stoploss mutations) or missense variants predicted to be damaging by at least four of the six pathogenic prediction tools. We included single-nucleotide (SNV) and Indel variations with a *variant fraction* >10% and coverage ≥20X (depth). This filtering led to the identification of approximately 3700 variants per trio. Among these, we focused on de novo variations and detected 37 heterozygous variants (Figure 1).

**Figure 2:**
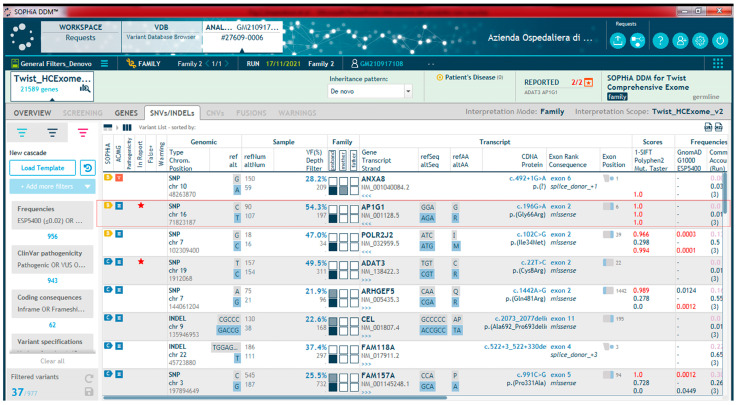
Table extracted from the Sophia DDM software (platform v5.10.42) analysis. The image shows part of the variant list obtained by sequencing and then filtering (placed on the left side of the figure). The red rectangle indicates the missense variant *AP1G1* c.196G>A, p.Gly66Arg, and some of its annotations.

Our analysis led to the identification of a heterozygous de novo missense variant in the *AP1G1* gene in the proband (Figure 2), which was confirmed by Sanger sequencing (Figure S1).

The c.196G>A (p.Gly66Arg) variant under investigation falls in exon 2 and has not been previously reported in the major human exome and genome sequencing public databases (GnomAD, https://gnomad.broadinstitute.org, accessed on 27 October 2025; Single Nucleotide Polymorphism database (dbSNP), https://www.ncbi.nlm.nih.gov/snp/, accessed on 27 October 2025; and Exome Sequencing Project (ESP5400), https://vatlab.github.io/vat-docs/applications/annotation/variants/esp/#:~:text=evs_5400%20NHLBI%20GO%20Exome%20Sequencing,the%20variant%20was%20found%20in, accessed on 27 October 2025).

### 2.2. In Silico Analysis of Variant Impact on Protein Structure and Function

**Figure 3:**
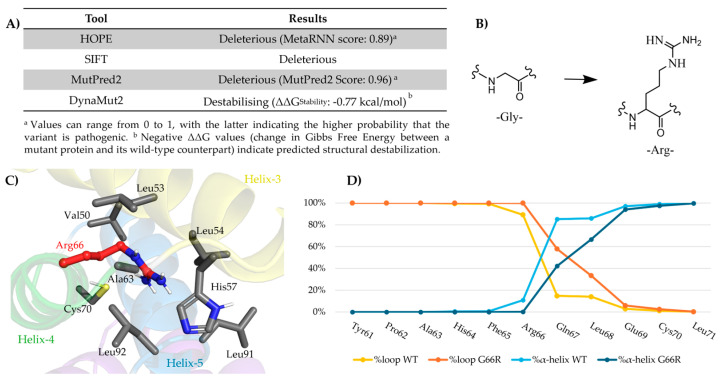
In silico analysis of the AP1G1 Gly66Arg variant. (**A**) Pathogenicity prediction using HOPE, SIFT, MutPred2, and DynaMut2, indicating a deleterious effect of the substitution. (**B**) Structural comparison of glycine and arginine. (**C**) Position of Arg66 (red sticks) and surrounding residues within 5 Å (gray sticks) in the AP1 complex model generated using AlphaFold. (**D**) Secondary structural organization of residues from Tyr61 to Leu71. For each residue, the percentage of secondary structure adopted during the molecular dynamics (MD) simulation was reported as the average across three replicas for both the wild-type (WT) and mutant (G66R) systems. The analysis included the percentage of loop (%loop) and α-helix (%α-helix) conformations.

The impact of the identified *AP1G1* variant was initially investigated in silico using multiple computational tools to predict the functional consequences of amino acid substitutions (Figure 3A). HOPE (https://www3.cmbi.umcn.nl/hope; accessed on February 2025), Sorting Intolerant from Tolerant (SIFT, https://sift.bii.a-star.edu.sg/; accessed on February 2025), and MutPred2 (http://mutpred.mutdb.org/#qform; accessed on February 2025) provided concordant results, all classifying the Gly66Arg substitution as pathogenic. Notably, HOPE and MutPred2 reported pathogenicity scores of 0.89 and 0.96, respectively, suggesting a high confidence in the deleterious nature of this variant. In parallel, DynaMut2 (https://biosig.lab.uq.edu.au/dynamut2/; accessed on February 2025) predicted a destabilizing effect of the mutation (ΔΔG_stability = −0.77 kcal/mol), indicating that the possible pathogenic effect of the single–amino acid genetic change is due to a structural perturbation of the protein organization. This predicted destabilization is consistent with the marked physicochemical differences between the two residues involved in the mutation (Figure 3B). Glycine is the simplest naturally occurring amino acid, characterized by a single hydrogen atom as its side chain, which accounts for its small molecular size. In contrast, arginine has a much larger positively charged guanidinium side chain, which is basic, highly polar, and capable of forming multiple hydrogen bonds and ionic interactions.

To explore the molecular basis of this effect, we generated three-dimensional structural models of the human AP1 complex, which consists of the σ, β, μ, and γ subunits, using AlphaFold tool (https://deepmind.google/technologies/alphafold/alphafold-server; accessed on February 2025). Two models were produced: a wild-type complex and a mutant complex, in which the Gly66Arg substitution was introduced into the γ-subunit. Structural inspection of the mutant revealed that the bulky guanidinium group of Arg66 was inserted between α-helices 3 (residues Tyr45-Gly60) and 5 (residues Phe79-Leu92) (Figure 3C; created using BioRender.com). The environment surrounding Arg66 is largely hydrophobic, rendering the presence of a charged arginine side chain chemically unfavorable. These observations provide a plausible explanation for the destabilizing effects predicted by DynaMut2 and gain further relevance, considering that this segment of the γ subunit is located near the interfaces involved in the inter-subunit assembly of the AP1 complex.

To assess the impact of the mutation under dynamic conditions, molecular dynamics (MD) simulations were conducted on both the wild-type and mutant AP1 complexes. Each system was simulated in triplicate for 500 ns to account for conformational variability differences between the two systems (Figure S2). Both complexes remained globally stable over time, and a localized analysis revealed structural differences in the residue object of the mutation. Indeed, the presence of Arg66 in the mutant—located at the beginning of α-helix 4 (residues Gly/Arg66-Ala75)—destabilized the helical structure and adopted a more flexible, loop-like configuration, an effect that also influenced the spatial arrangement of the adjacent residues Gln67 and Leu68 (Figure 3D). Notably, the observed local structural transition indicates mutation-induced destabilization, which likely impairs the local folding landscape and reduces the compatibility with neighboring subunits. These findings suggest that the substitution at residue 66 may hinder proper subunit assembly and compromise overall protein stability, offering mechanistic insights into its predicted pathogenic effects.

### 2.3. Immunofluorescence of WT and c.196G>A Fibroblasts

To gain further insights into the possible pathogenic effect of this de novo variant (p.Gly66Arg) and to reach a diagnosis for the patient, we performed a skin biopsy to obtain fibroblast cultures, which were useful for in vitro analysis of the functional effect of the *AP1G1* variant. To determine the subcellular localization of wild-type and mutant AP1G1 proteins, immunofluorescence staining was performed using antibodies targeting intracellular markers. GM130 is a cis-Golgi protein involved in the structural organization of the Golgi apparatus and tethering of vesicles from the endoplasmic reticulum. TGN46 is a marker of the trans-Golgi network that is implicated in the sorting of proteins into endosomal compartments and the plasma membrane. AP1G1 is a subunit of the AP-1 adaptor complex responsible for the recognition of protein cargo and the formation of clathrin-coated vesicles between the TGN and endosomes.

**Figure 4:**
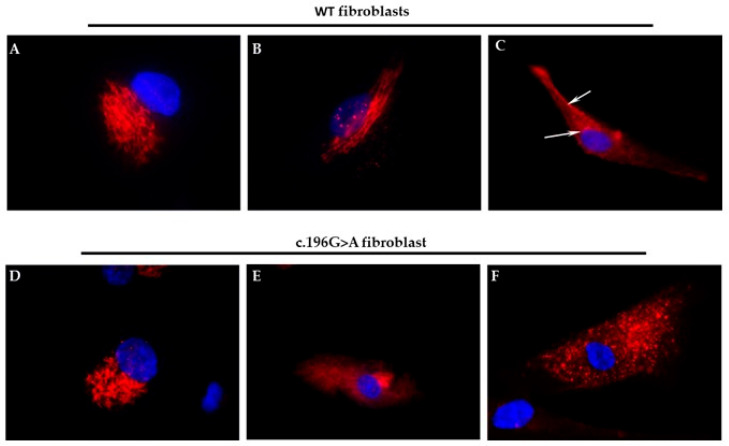
Subcellular localization of Golgi markers and AP1G1 in WT and c.196G>A mutant fibroblasts. (**A**) The cis-Golgi marker GM130 exhibited a typical compact perinuclear localization, consistent with an intact cis-Golgi structure. (**B**) The trans-Golgi network marker TGN46 exhibited a focused perinuclear pattern, outlining the organized architecture of the trans-Golgi cisternae. (**C**) AP1G1 was localized to the internal and peripheral regions of the cellular membrane, as well as in the perinuclear area (indicated by arrows), consistent with its role in vesicle formation in the trans-Golgi network and endosomes. (**D**) GM130 maintained a perinuclear distribution similar to that of WT, indicating that the overall organization of the cis-Golgi was preserved in the mutant cells. (**E**) TGN46 labeling appeared more dispersed and less defined, suggesting disorganization of the trans-Golgi network architecture in the presence of the mutation. (**F**). AP1G1 showed a punctate cytoplasmic distribution and was notably absent from membrane-associated regions, indicating defective membrane recruitment, likely resulting from impaired function of the mutant protein. In all panels, the nucleus is shown in blue via DAPI staining.

In WT fibroblasts, immunolabeling of the Golgi apparatus and trans-Golgi network with GM130 (Figure 4A) and TGN46 (Figure 4B) antibodies, respectively, revealed the expected localization to the Golgi cisternae and associated vesicles. In contrast, AP1G1 labeling was observed on both internal and peripheral cellular membranes.

In c.196G>A mutant fibroblasts, although GM130 labeling remained comparable to that in the controls, the organization of the trans-Golgi cisternae appeared more diffuse and disorganized. Notably, AP1G1 displayed a punctate cytoplasmic distribution and was markedly absent from the cellular membranes. The observed phenotype is likely due to a dominant-negative effect or a toxic gain-of-function of the mutant *AP1G1*, impairing the activity or recruitment of the wild-type protein. In conclusion, the c.196G>A mutation in *AP1G1* disrupts membrane association and impairs vesicular trafficking at the trans-Golgi level, potentially resulting in significant functional consequences.

**Figure 5:**
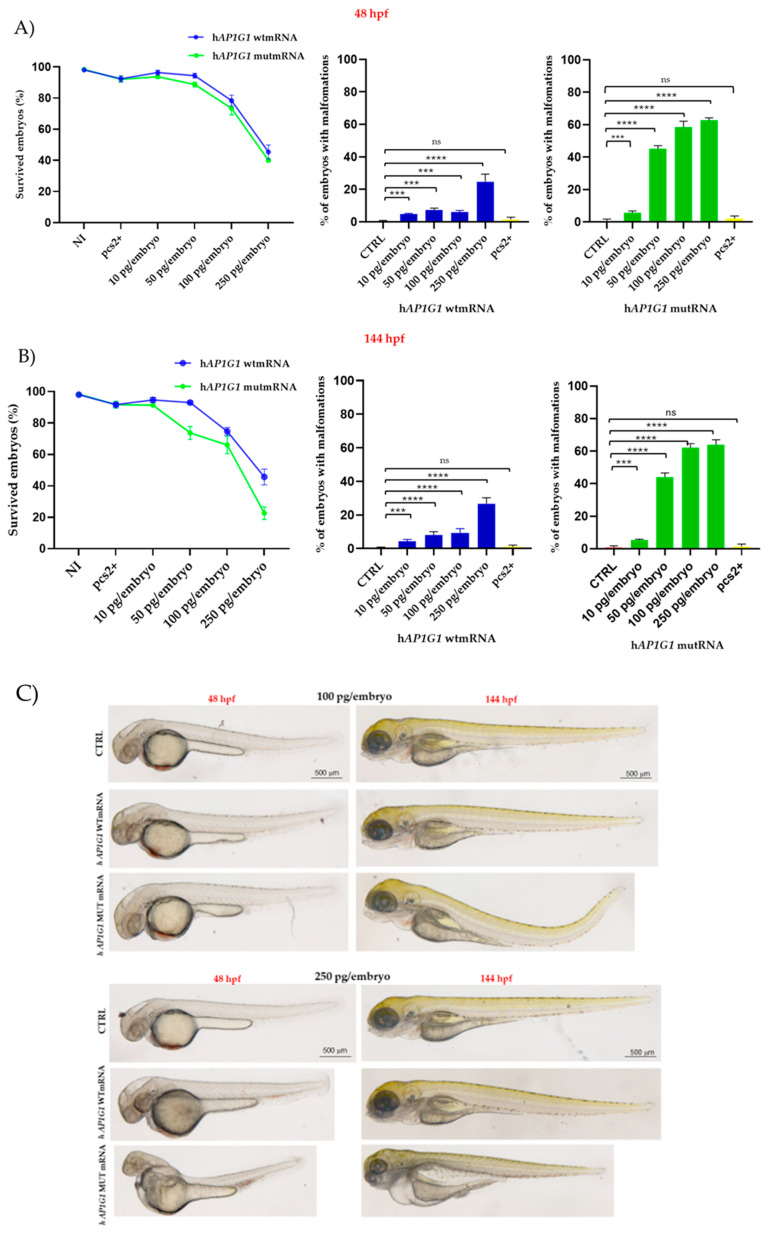
Survival and malformation rates of embryos injected with human *AP1G1* WT and MUT mRNA. The *X*-axis shows the survival rate (%) of embryos, while the *Y*-axis shows the mRNA concentration injected in a final volume of 4 nl at the following concentrations: 10, 50, 100, and 250 pg/embryo. CTRL (−) represents not-injected-embryos. At the same developmental stages, the morphology of embryos injected with human *AP1G1* WT or MUT mRNA was carefully evaluated by visual microscopic inspection, considering changes in brain regions, tail morphology, the presence of pericardial edema, and notochord malformations. (**A**,**B**) Survival and malformations were analyzed at 48 and 144 hpf. (**C**) In Figure 5C are shown representative embryos at both developmental stages and injected with 100 and 250 pg/embryo of both human WT and MUT *AP1G1* mRNA. The results represented in (**A**–**C**) panel are expressed as the mean ± SD of four independent experiments, with 30 embryos for each experiment and treatment. (*** *p* < 0.001 vs. control group; **** *p* < 0.0001 vs. control group; ns = not significant).

To investigate the in vivo effects of the AP1G1 mutation during neurodevelopment, we overexpressed human WT and mutant mRNAs in zebrafish embryos. To establish the optimal mRNA dose for subsequent experiments, a dose–response analysis was performed for both mRNAs. Initial testing evaluated doses of 10, 50, 100, and 250 pg/embryo. Non-injected embryos and embryos injected with plasmid pCS2+ were used as controls for all experiments. Injections were performed at the one-cell stage, and the survival rate was evaluated at 48 hpf (Figure 5A). More than 98% (*n* = 117/120) of the non-injected embryos survived, whereas 96% (*n* = 115/120) and 93% (*n* = 111/120) of the embryos survived when injected with 10 and 50 pg/embryo of mutant mRNA, respectively. Additionally, embryo survival decreased to 78% (*n* = 92/120) and 55% (*n* = 54/120) when injected with 100 pg/embryo and 250 pg/embryo of mutant mRNA, respectively. These survival rates were comparable to those observed when embryos were injected with the same dose of human WT *AP1G1* mRNA (blue line). Next, we evaluated malformations in embryos injected with either wild-type or mutated mRNAs (hereinafter abbreviated as WT and MUT, respectively). Morphological deformities were observed in 40% of the embryos injected with 50 pg/embryo MUT mRNA (*n* = 48/120). Embryos injected with MUT mRNA exhibited a dose-dependent increase in morphological deformities, with abnormality rates of 58% (70/120) at 100 pg/embryo and 62% (75/120) at 250 pg/embryo. Embryos injected with human WT *AP1G1* mRNA exhibited morphological changes at a markedly lower frequency than non-injected embryos. Survival and malformation rate analysis was also performed on injected embryos at 144 hpf, but no dramatic changes were observed, and the results are shown in Figure 5B. Based on these findings, 50 pg was selected as the optimal dose for both mRNAs in subsequent experiments as it yielded >80% survival and <20% malformation rates.

### 2.4. Rescue of the Lethal Phenotype of Ap1g1−/− Embryos by Injection of Human WT and MUT AP1G1 mRNAs

**Figure 6:**
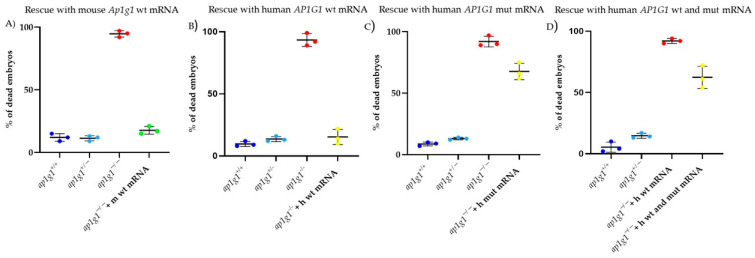
Dominant-negative effect of human *AP1G1* MUT mRNA. Graphs show the percentage of dead embryos at 48 hpf for the three different genotypes and the percentage of dead embryos after microinjection of (**A**) mouse *Ap1g1* WT mRNA, (**B**) human *AP1G1* WT mRNA, (**C**) human *AP1G1* MUT mRNA, and (**D**) co-injection of human *AP1G1* WT/MUT mRNA into *ap1g1−/−* knockout embryos. Each experiment was repeated three times, and 30 embryos were injected for each condition.

In a previous study, we obtained a substantial rescue of the survival rate of *ap1g1−/−* knockout embryos at 48 hpf [24] when injected at the one-cell stage with WT *Ap1g1* mouse mRNA (Figure 6A). In the current study, we performed analogous rescue experiments using both WT and MUT human *AP1G1* mRNAs. As shown in Figure 6B, the injection of 50 pg/embryo of human *AP1G1* WT mRNA was as effective as the mouse counterpart in restoring the viability of mutant embryos, whereas the survival rate drastically decreased when a dose of 50 pg/embryo of *AP1G1* MUT mRNA was injected (Figure 6C). Based on a dose-curve, the co-injection of both WT and MUT *AP1G1* mRNAs in a ratio of 25/25 pg per embryo, respectively, only slightly improved the survival of the injected embryos compared to the injection of MUT *AP1G1* alone (Figure 6D).

### 2.5. Morphological Assessment During Embryonic Development of Embryos Expressing Human WT or MUT AP1G1 mRNA

We injected zebrafish embryos at the one-cell stage with either WT or MUT *AP1G1* mRNAs at the optimal selected dose of 50 pg/embryo. Morphological parameters, including abnormal body shape, central nervous system abnormalities, pericardial edema, tail deformities, and notochord, were assessed at 24, 48, 72, and 144 hpf (Table 1).

**Figure 7:**
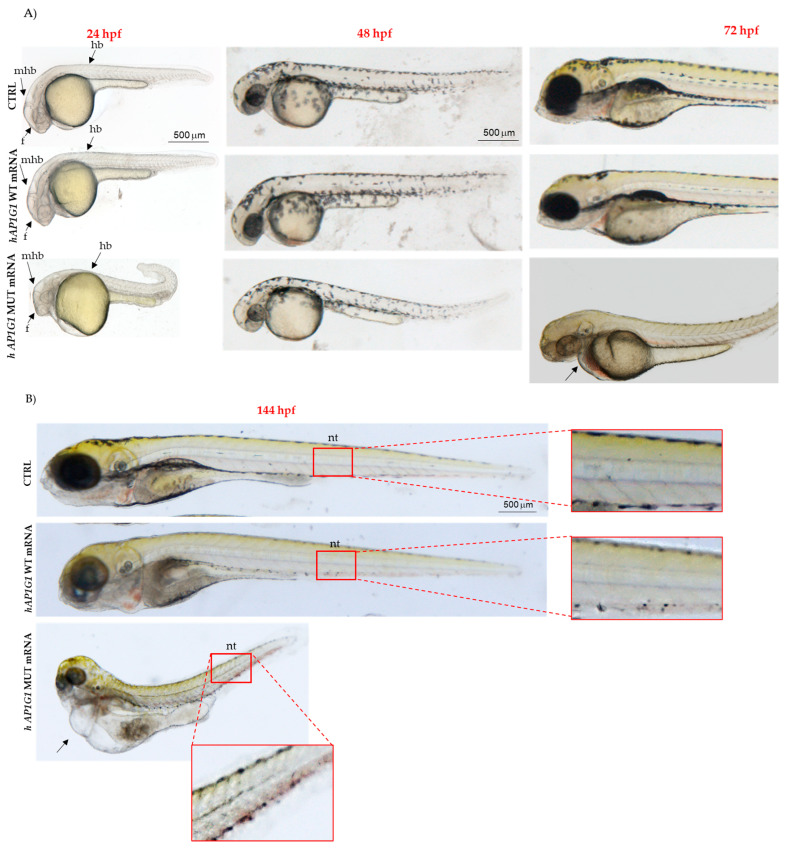
Morphological phenotypes of embryos injected with human WT and MUT *AP1G1* mRNA. (**A**) Representative embryos at 24, 48, 72, and 144 hpf developmental stages showing the main morphological effects after the injection of human WT and MUT *AP1G1* mRNAs. Experiments were conducted three times, and 40 embryos were injected with either mRNAs. Uninjected embryos were used as controls (CTRL group). All images are lateral views with the anterior to the left (magnification 20×). (**B**) 144 hpf. Arrow indicates edema. Abbreviations: f, forebrain; mhb, midbrain–hindbrain boundary; hb, hindbrain; nt, notochord that are indicated by arrows in Figure 7A.

Despite the low mortality at the selected concentration, a significant proportion (42%) of embryos injected with human MUT *AP1G1* mRNA exhibited a growth delay, characterized by shorter body length and a curved tail. Neither significant alterations nor growth delays were observed in embryos injected with WT mRNA or in non-injected embryos (Figure 7A). At 48 hpf, morphological abnormalities in the central nervous system (CNS) were observed in 40% (*n* = 48/120) of the embryos injected with human MUT *AP1G1* mRNA. Phenotypic severity increased at later stages, with 33% (*n* = 39/120) and 53% (*n* = 64/120) of the embryos exhibiting pronounced abnormalities at 72 and 144 hpf, respectively. Notably, embryos injected with human MUT *AP1G1* mRNA exhibited phenotypic alterations, including abnormal somite morphology, anterior–posterior axis malformations, and pericardial edema. All human MUT *AP1G1* mRNA-injected embryos were compared with both WT *AP1G1* mRNA-injected embryos and non-injected controls (Figure 7B).

### 2.6. Transient Expression of Human MUT AP1G1 mRNA Induces Abnormal Behavior in Zebrafish Embryos

**Figure 8:**
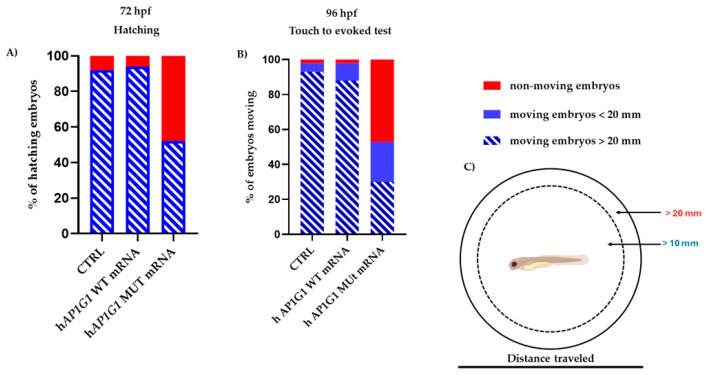
Hatching and touch-evoked tests to assess locomotor responses. Zebrafish embryos were injected with human WT and MUT *AP1G1* mRNA, and hatching rate and touch-evoked tests were performed at 72 and 96 hpf, respectively. Three independent replicates were performed, with 30 embryos for each experiment and treatment (90 embryos per condition). Non-injected embryos were used as controls (CTRL). (**A**) Percentage of embryos hatched at 72 hpf. (**B**) Embryos were injected with human WT and MUT *AP1G1* mRNAs and maintained at 28 °C until 96 hpf. After gently touching the tail of each embryo, the touch swimming response was observed and categorized as follows: embryos that did not move, those that swam < 20 mm within the motility wheel, and those that swam > 20 mm in the wheel. Percentages of each category for each mutant were then calculated, considering 30 embryos for each treatment and each of the three experiments, and plotted (**C**) Schematic representation of the motility wheel used for the touch-evoked test.

Zebrafish is a valuable vertebrate model for studying the relationship between the central nervous system and behavior. Its brain exhibits a relatively simple yet conserved structural organization and cellular morphology, closely resembling that of other vertebrates, including chickens, rats, and humans [27,28]. Notably, midbrain catecholinergic neurons are dopaminergic and homologous to their mammalian counterparts. These neurons, which are critical for motor control regulation, share structural and functional similarities with those found in the mammalian midbrain structures. The first movements of zebrafish embryos consist of spontaneous contractions of the tail, which begin at 17 hpf. These early movements coincide with the presence of primary mechanosensory neurons in the spinal cord of the embryo. In zebrafish embryos, hatching from the chorion is a physiological process that occurs between 48 and 72 hpf, is strictly correlated with neurodevelopment, and depends on tail movements [29,30]. Taking advantage of these physiological traits, we assessed motor responses and behavioral differences in embryos injected with human WT or MUT *AP1G1* mRNAs using two different tests. First, we measured the “hatching rate,” observing that embryos injected with MUT *AP1G1* mRNA showed a significantly reduced hatching rate (50%) compared to WT-injected and non-injected controls (CTRL) (Figure 8A).

We then investigated whether the injection of either *AP1G1* mRNAs could alter the somatosensory system. To assess this, we performed a touch-evoked response assay at 96 hpf by gently stimulating the tail with a pipette tip and measuring the swimming distance after the stimulus was categorized as <20 mm or >20 mm) [31]. Nearly all (95%) non-injected embryos and those injected with WT *AP1G1* RNA swam > 20 mm, whereas only a small fraction exhibited limited (<20 mm; 4%) or no movement (3%). In contrast, 50% of the embryos injected with human *AP1G1* MUT mRNA failed to respond to the touch stimulus, 23% had a reduced response (<20 mm), and only 27% displayed normal escape behavior (>20 mm) (Figure 8B). These results suggest that the transient expression of MUT *AP1G1* mRNA compromises both spinal motor neuron function and mechanosensory processing, leading to defective locomotor behavior in embryos.

**Figure 9:**
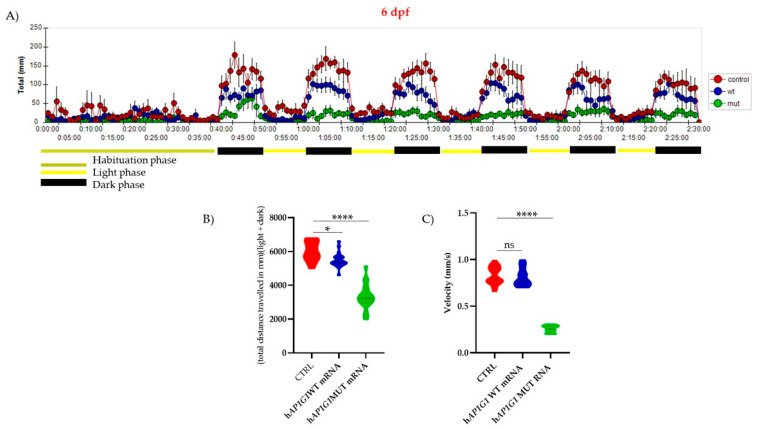
Behavioral analyses of larvae injected with either WT or MUT human *AP1G1* mRNAs and compare to not injected embryos (CTRL). (**A**) Time course of the distance traveled by the larvae (6 days post-fertilization) overexpressing either WT or MUT human *AP1G1* mRNAs and not injected embryos during 120 min of observation, with the first 30 min spent in the light (habituation phase). Each data point represents the mean (±SEM) of all different subjects (WT *AP1G1* mRNA *n* = 50, MUT *AP1G1* mRNA *n* = 48; CTRL *n* = 50). The experiment was performed in triplicate. Locomotor activity was quantified as the total distance traveled by the larvae (cm) (**B**) and their velocity (mm/s). (**C**) The Results in (**B**,**C**) are expressed as violin plot distributions: inside each violin, the central dashed line marks the median (Q2), and the superior and inferior dotted lines mark the third (Q3) and first (Q1) quartiles, respectively. The raw data plot represents the mean ± SD of the distance traveled by embryos in 2 min time bins of three experiments performed with the Noldus *Ethovision* software (XT 13.0 version). Data from independent experiments were analyzed using one-way ANOVA with Tukey’s multiple comparison test: * *p* < 0.005; **** *p* < 0.0001; ns = not significant.

Zebrafish larvae exhibited mature swimming at 4 dpf as a consequence of swim bladder development and proper neurodevelopment. Locomotor activity also exhibits differential responses to lighting conditions: light-dark transitions increase activity, whereas dark-light transitions reduce it [28]. To assess whether WT or MUT *AP1G1* expression influences mature swimming behavior, we injected zebrafish embryos with the respective mRNAs at the one-cell stage. Larvae were grown until 6 dpf, and their motor behavior was evaluated using an automated motion-tracking system. We first assessed general swimming behavior under light conditions for 90 min, followed by 30 min in the dark, using the Noldus *DanioVision* chamber. This system enables individual video tracking of larvae in a 24-well plate arena. The results revealed that embryos injected with human MUT *AP1G1* RNA exhibited significant reductions in the total distance traveled and rapidity of movement compared to WT *AP1G1* RNA-injected embryos and not injected embryos (Figure 9). Moreover, there was a significant reduction in velocity in MUT *AP1G1* RNA-injected embryos (Figure 9), with no significant difference in active time compared to embryos injected with WT *AP1G1* RNA and not injected embryos. These findings indicate that transient expression of MUT *AP1G1* RNA significantly perturbs the behavior of zebrafish embryos.

### 2.7. Expression of Human MUT AP1G1 mRNA Alters Development of Specific Brain Regions of Zebrafish Embryos

**Figure 10:**
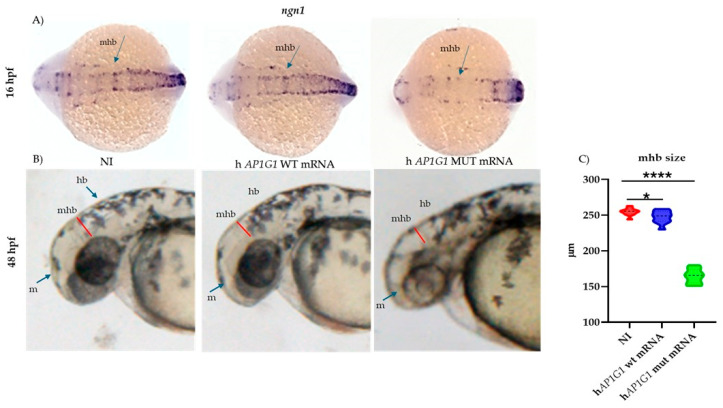
Expression of MUT *AP1G1* mRNA affects midbrain–hindbrain boundary formation. Expression of zebrafish neurogenin 1 (*ngn1*) during early development in the midbrain–hindbrain boundary (mhb) of embryos injected with either human WT or MUT *AP1G1* mRNA. All treatments, including the controls (non-injected embryos), were performed in triplicate, with 30 embryos per condition (90 embryos per experimental group). The embryos shown in the figure are representative of all experiments. (**A**) Dorsal views (anterior to the left) of 16 hpf embryos at 25× magnification. (**B**) Lateral views (dorsal to the top and anterior to the left, 25× magnification) of 48 hpf embryos. (**C**) Violin plot distribution of midbrain–hindbrain boundary (mhb) measurements at 48 hpf for non-injected and injected embryos Measurements were performed on digital images using the ImageJ software (Version 1.8.0). The *Y*-axis shows the mhb length (μM). Measurements were performed on 10 embryos for each of the three independent experiments and for each treatment (a total of 30 embryos for each experimental condition). Inside each violin, the central dashed line marks the median (Q2), and the superior and inferior dotted lines mark the third (Q3) and first (Q1) quartiles, respectively. Measurements were analyzed among and between groups using one-way ANOVA with post hoc Tukey’s multiple comparison test (* = *p* < 0.05; **** = *p* < 0.0001). (h, hindbrain; m, midbrain; mhb, midbrain–hindbrain boundary).

Given the close association between CNS development and behavior, we sought to identify the key brain regions underlying the observed phenotypic differences in embryos injected with either WT or MUT *AP1G1* mRNAs. We performed whole-mount in situ hybridization (WISH) analysis of *ngn1*, a transcription factor expressed at the onset of neurogenesis in the zebrafish neural plate and a key marker of neuron differentiation. The results showed that 16 hpf zebrafish embryos injected with human MUT *AP1G1* mRNA displayed poorly defined major brain structures and a malformed or absent midbrain–hindbrain boundary compared to non-injected controls or embryos expressing WT *AP1G1* mRNA (Figure 10). Quantitative analysis at 48 hpf revealed severe structural abnormalities of the MHB in MUT *AP1G1* mRNA-injected embryos, confirming pronounced neurodevelopmental disruption compared with controls (Figure 10).

**Figure 11:**
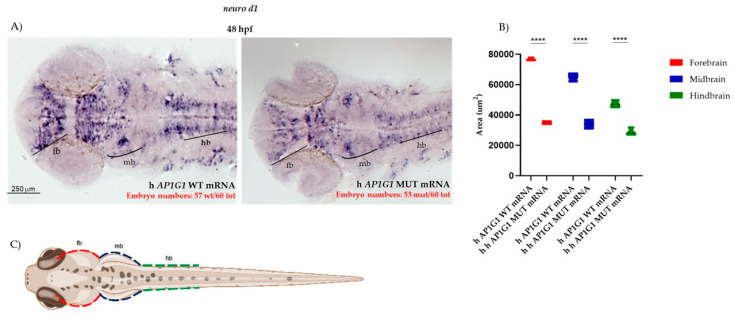
Human *AP1G1* MUT mRNA expression induces neuronal defects during early embryonic development. (**A**) Expression of *ngn1* at 48 hpf was analyzed by whole-mount in situ hybridization (WISH) in embryos injected with either human WT or MUT *AP1G1* mRNA. Representative images from three independent experiments, with 20 embryos per condition in each replicate (total number of embryos with the described phenotype is shown in red for each experimental condition). Dorsal views are shown at 20× magnification. Abbreviations: fb, forebrain; hb, hindbrain; mb, midbrain. (**B**) The graphic shows measurements for the forebrain, midbrain and hindbrain area analyzed using ImageJ software (Version 1.8.0). (**C**) The drawing represents the areas (colored differently) that were identified for the measurements. Three distinct experiments were performed, with 10 embryos per treatment (30 embryos for each experimental condition) considered for analysis. Data are presented as violin plots. Inside each violin, the central dashed line marks the median (Q2), and the superior and inferior dotted lines mark the third (Q3) and first (Q1) quartiles. Statistical significance among and between groups was calculated using one-way ANOVA with post hoc Tukey’s multiple comparison test (**** = *p* < 0.0001).

In embryos injected with human WT *AP1G1* mRNA, *ngn1* was expressed at 48 hpf in several brain regions, such as the forebrain (telencephalon and anterior and posterior diencephalon), midbrain, hindbrain, and along the spinal cord, where both motor neurons and primary mechanosensory neurons reside (Figure 11). Microinjection of human MUT *AP1G1* mRNA induced an evident reduction in the areas of *ngn1* expression in all the main brain and spinal cord regions, as shown in the dorsal view of the embryos. Interestingly, human MUT *AP1G1* mRNA expression also resulted in decreased *ngn1* expression in the ventral and dorsal diencephalon, where the earliest dopaminergic neurons are normally detected (Figure 11).

**Figure 12:**
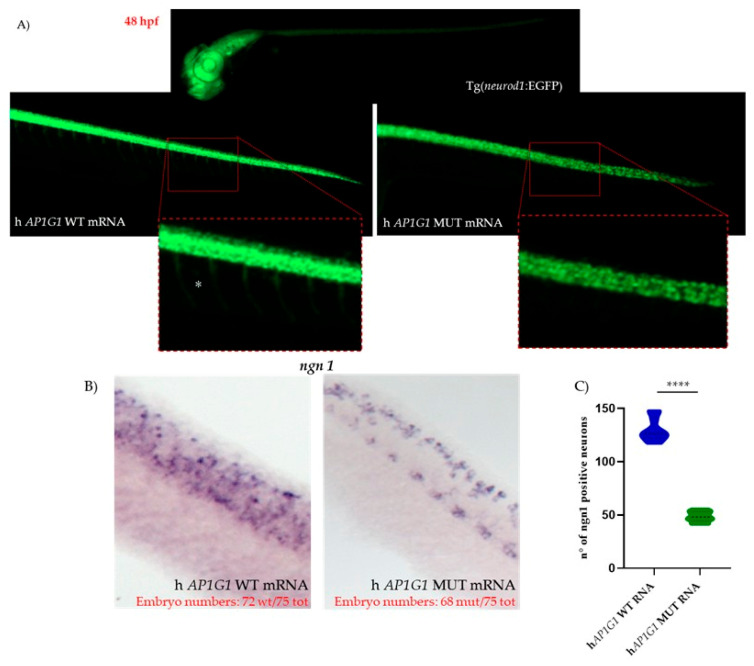
Expression of human MUT *AP1G1* mRNA resulted in a strong reduction in *nrd1*-positive motoneurons in 48 hpf embryos. (**A**) *nrd1*-dependent EGFP fluorescence in zebrafish embryos after injection of either WT or MUT *AP1G1* mRNAs. Representative images (obtained with a fluorescence microscope) of three independent experiments with 25 embryos per condition in each replicate (total number of embryos with phenotypes is shown in red numbers for each experimental condition in panel (**B**). Lateral views of the head and spinal cord regions (20× magnification) are shown, with further magnification (40×) for the region of the spinal cord in the inset in three distinct experiments and in 10 embryos per treatment (a total of 30 embryos for each experimental condition). (**B**) Lateral view of a segment of the spinal cord from the WISH experiment performed with the *ngn1* probe on zebrafish embryos after injection of either WT or MUT *AP1G1* mRNAs. (**C**) Violin plot distribution of *ngn1*-positive neurons. The central dashed line marks the median (Q2), and the superior and inferior dotted lines mark the third (Q3) and first (Q1) quartiles. To quantify the number of neurons, statistical significance among and between groups was calculated using one-way ANOVA with post hoc Tukey’s multiple comparison test (**** = *p* < 0.0001).

To further evaluate the effects of MUT *AP1G1* mRNA expression on zebrafish neurogenesis, we used a transgenic zebrafish line expressing Enhanced Green Fluorescent Protein (EGFP) under the control of the *neurod1* promoter, referred to as Tg(*nrd1*:EGFP). *nrd1* is a basic helix–loop–helix (bHLH) transcription factor that acts downstream of *ngn1*, and it is expressed in differentiated neurons of various brain areas as well as in the trunk motoneurons [32]. At 48 hpf, embryos injected with human MUT *AP1G1* mRNA exhibited a strong reduction in *nrd1*-positive motoneurons compared to embryos injected with human WT *AP1G1* mRNA (Figure 12). Time-lapse imaging from 18 to 24 hpf acquired via Light Sheet Fluorescence Microscopy in the Tg(*nrd1*:EGFP) line injected with either WT or MUT *AP1G1* mRNA corroborated prior findings (Supplementary Video). This analysis highlighted distinct differences in *neurod1*-positive neurons across the experimental groups.

## 3. Discussion

Ubiquitin adaptor protein complexes (AP) mediate selective intracellular vesicular trafficking and polarized localization of somato-dendritic proteins in neurons. AP complexes play a key role in the trafficking and correct localization of cargos in different cell types, particularly neurons. Notably, most patients with disorders related to defects in the subunits of AP complexes exhibit abnormalities in brain function and structure, likely due to the complex organization of neurons and glial cells, which have very long differentiated axons that increase their vulnerability to defects in intracellular transport. Disease-causing mutations have been found in *all* AP complexes, including AP-1 and AP-2, which are essential for vertebrate survival.

The lack of an AP subunit renders the entire complex non-functional [6]; therefore, loss-of-function mutations in any subunit of a particular complex should have the same phenotype. A substantial part of our understanding regarding the function of AP-1 complex has been derived from studies employing *knockout* and *knockdown* techniques. However, recent evidence from animal models and human patients indicates that point mutations in ubiquitously expressed AP-1 subunits can lead to distinct diseases collectively known as *adaptinopathies*. Mice homozygous for a null mutation of the AP-1 γ1 subunit die at day 3.5 post-fertilization, while heterozygous mice display a reduced growth rate exclusively during nursing and impaired T-cell development [19]. In murine models, the loss of AP-1 complex subunits is associated with prenatal lethality or severe growth deficits, and disease-causing alleles of various subunits of AP complexes have been implicated in several human disorders, such as intellectual disabilities (IDs). In 2024, Zhang J. and his colleagues reported that pathogenetic variants in 13 genes encoding different subunits, of the 5 AP complexes, are associated with several neurodevelopmental disorders; the data suggested the important role of AP complexes in neurodevelopment and neuronal functions [33].

The important and essential role of the AP-1 complex in correct development and cognitive function in humans has been demonstrated. In 2021, 11 unrelated patients were described with mild to severe intellectual disability, speech delay, and aggressive behavior in 100% of the patients, while other aspects (including autism, epilepsy, hypotonia, spasticity, and limb defects) were variably expressed in the cohort [1]. In 2024, another missense variant that appeared to be correlated with specific facial features was identified [2].

Our proband showed a novel missense variant in the amino-terminal region involving a glycine-conserved residue that was substituted with an arginine. The normal final folding of the protein results in compression due to the substantially different properties of the two amino acids, leading to a dysfunctional protein. In particular, the chemical structure of glycine is characterized by a neutral charge and apolarity with a high facilitation of local folding and movements, whereas arginine is a positive polar amino acid with a rigid local structure. These findings indicated that the variant was responsible for the girl’s phenotype.

To date, *all* reported pathogenic missense variants are located within the N-terminal region and involve highly conserved residues. All functional studies in correlation with the clinical phenotype of all patients suggested that these de novo heterozygous variants could have a toxic dominant-negative effect on biological mechanisms.

To further support this hypothesis, we performed a comparative analysis of clinical and zebrafish phenotypes across all reported N-terminal AP1G1 missense variants, including our novel p.Gly66Arg. Patients carrying already reported p.Arg15Gln, p.Arg35Trp, and p.Arg35Gln variants consistently presented with global developmental delay, intellectual disability, speech delay, and behavioral anomalies, including aggression and, variably, seizures, hypotonia, and autistic traits. Our proband exhibited a highly overlapping clinical profile, reinforcing the phenotypic convergence within this mutational cluster.

Functional studies in zebrafish further corroborate this interpretation.

Complementing rodent models, zebrafish have emerged as a valuable model organism for CNS research and related diseases. The suitability of zebrafish as an in vivo model for investigating rare neurodevelopmental disorders is further corroborated by the fact that approximately 80% of the human disease genes are conserved in *Danio rerio*. This conservation of protein-coding sequences enables the functional analysis of individual mutations, thereby shedding light on pathogenic mechanisms and potentially guiding therapeutic exploration. Moreover, the external development of zebrafish embryos, combined with their optical transparency and the availability of transgenic lines expressing EGFP in distinct neuronal populations, enables direct real-time visualization of neuronal phenotype development and organization. This approach provides researchers with deeper insights into the mechanisms underlying neurodevelopmental diseases [29]. It is well known that there are high anatomical similarities between zebrafish and the human nervous system, particularly in its main structures such as the hippocampus, diencephalon, tectum, and cerebellum [34]. Additionally, zebrafish dopaminergic neurons located in the diencephalon are homologous to mammalian midbrain catecholaminergic neurons, which play a crucial role in motor behavior. The projection of dopaminergic neurons is closely related to the regulation of certain behaviors [35]. Finally, with the help of genome engineering technologies, including DNA editing technology (CRISPR/Cas9), next-generation DNA sequencing, transgenesis, and overexpression of both wild-type and mutated mRNA, it is possible to replicate the pathogenic phenotype associated with mutations identified in human patients. To further investigate the pathogenic mechanism of the identified variant, we injected one-cell-stage embryos with both WT and MUT *AP1G1* mRNAs. The most notable findings were morphological developmental abnormalities characterized by shorter body length, pericardial edema, abnormal body shape, notochord, and tail deformities, which led us to infer a strong effect of human mutant *AP1G1* mRNA expression. In particular, at 48 hpf, a strong neuronal phenotype, including brain abnormalities, was observed in embryos injected with human MUT mRNA. These morphological alterations were accompanied by significant behavioral deficits, as evidenced by decreased total swimming distance, average speed, and movement time.

Among vertebrate models, zebrafish provides a special opportunity to connect brain circuit activity with behavior. The first movements of the larva, characterized by spontaneous tail contractions, appear at 17 hpf [30]; at this stage, only six types of spinal neurons are active, with a repeating segmental organization [36]. Touch-evoked responses mediated by Rohon-Beard sensory neurons were detectable at 21 hpf. By 48–72 hpf, fish are able to swim in a coordinated manner and respond appropriately to different stimuli [30]. Given the well-established link between neural activity and motor behavior, our study explored the potential role of *ap1g1* during crucial phases of embryonic neurodevelopment using simple motor and behavioral assays. In zebrafish, the hatching period typically occurs between 48 and 72 hpf and represents the transition point from a developing embryo to a free-living larva. This process is tightly regulated by many factors and is highly sensitive to the first motor movements in response to midbrain neurons. Embryos injected with human MUT *AP1G1* mRNA showed a strong reduction in hatching rate compared to embryos injected with human WT *AP1G1* mRNA. The spinal cord of zebrafish embryos harbors primary mechanosensory neurons that play a critical role in the early development of the somatosensory system. At 72 hpf, we examined whether human mRNA injection affected somatosensory function. To assess this, we performed a touch-evoked test by gently touching the tail with a pipette tip and recording the distance swum following the stimulus. Responses were classified based on the total distance swum using a threshold of 20 mm to distinguish between reduced (<20 mm) and normal or enhanced (>20 mm) escape behaviors. Control embryos and embryos injected with human WT *AP1G1* mRNA were able to travel a distance > 20 mm, in contrast to a large percentage of embryos injected with human MUT *AP1G1* mRNA, which did not respond to touch stimuli. These findings suggest that transient expression of human MUT *AP1G1* mRNA may affect spinal motor neuron function, impacting embryo locomotor behavior. Furthermore, we studied their swimming performance at 144 hpf after injection of both *AP1G1* mRNAs, considering that at this stage the neurogenesis process is already complete. Using an animal motion-tracking system (*EthoVision XT*), we quantified the total swimming distance under alternating light and dark conditions. Zebrafish larvae injected with WT *AP1G1* mRNA displayed normal locomotor activity, whereas larvae injected with MUT *AP1G1* exhibited a marked reduction in swum distance. Collectively, these findings indicate that the c.196G>A (p.Gly66Arg) mutation in *AP1G1* significantly alters the behavior of zebrafish embryos.

The midbrain–hindbrain boundary (MHB) serves as a critical organizing center during vertebrate brain development. This structure is composed of motor neurons that regulate eye, head, and body movements and plays an essential role in sensory-motor integration. Furthermore, the MHB acts as a key signaling hub required for patterning and neural differentiation—particularly of *ngn1*-positive neurons—in the midbrain and anterior hindbrain [37]. One of the most crucial genes involved in early embryonic neurodevelopment and neuronal commitment is *neurogenin 1* (*ngn1*), a neural-specific basic helix-loop-helix (bHLH) transcription factor. *ngn1* is expressed at the onset of neurogenesis in zebrafish embryo neural plate domains that give rise to sensory neurons of the dorsal root ganglia and primary motor neurons that control embryo motility [38]. Using the WISH technique, we observed that the injection of mutant mRNA caused the downregulation of *ngn1* mRNA expression at the early stages of development. This reduction may explain the initial phenotypic alterations observed, particularly delayed or impaired hatching rates. *ngn1* is also required for the development of sensory neurons in the zebrafish trunk. It is expressed early in the Rohon-Beard spinal sensory neurons, located within the spinal cord, and later in the dorsal root ganglia. These sensory neurons are considered touch-responsive neurons that induce locomotion following a tactile stimulus, a form of escape behavior [39]. Following the injection of human MUT *AP1G1* mRNA, *ngn1* expression was lower in spinal cord neurons, where primitive sensory neurons reside, and in the main brain regions analyzed, such as the diencephalic area (where dopaminergic neurons are located), midbrain, and hindbrain, compared to embryos injected with human WT mRNA. Our findings suggest that mutations in the human *AP1G1* gene might also affect the somatosensory system; the observed locomotor impairments could be attributed not only to altered sensory neuron development but also to dysfunction in dopaminergic and motor neurons, as previously discussed. These findings are consistent with previously published results obtained from different in vivo models, in which the removal of one of the subunits belonging to different complexes reduced motor coordination or caused behavioral disturbances [40].

Finally, we were able to rescue the mortality rate of *ap1g1−/−* knockout embryos (mutant line generated by our group [24]) when we injected the human WT *AP1G1* mRNA; in contrast, embryos injected with human MUT *AP1G1* mRNA exhibited a significantly increased mortality rate. Co-injection of both mRNAs resulted in a slight increase in the survival rate, highlighting the dominant-negative effect exerted by the human MUT mRNA.

In conclusion, the use of zebrafish not only contributes to elucidating the mechanisms underlying neurodegenerative diseases but also holds promise for identifying genetic and molecular targets to develop effective therapeutic strategies in the context of adaptinopathies.

## 4. Materials and Methods

### 4.1. Samples and DNA Extraction

We conducted a “*Whole-Exome Sequencing project*” funded by the Italian Health Ministry (SG-2018-12368345 to V.I.). Patients were enrolled from a cohort of individuals showing intellectual disability and/or autism and/or congenital malformation syndromes who were referred to the Medical Genetics Unit of Perugia’s Hospital (Italy) for genetic counseling and remained undiagnosed after several negative genetic tests. Informed consent for the study was obtained, and genomic DNAs (gDNAs) of the proband and her parents were isolated from EDTA peripheral blood samples using a QIAamp DNA Blood Kit according to the manufacturer’s protocol (Qiagen, Hilden, Germany).

### 4.2. Case Presentation

The patient, currently a 12-year-old girl, was born to healthy, non-consanguineous parents following an uncomplicated pregnancy and delivery (Figure S1). She exhibited motor and speech delays, achieved independent walking at 18 months, and pronounced fewer than 10 words at 36 months. At the age of 2 years, she experienced a single febrile seizure, and subsequent EEG and polysomnography results were normal. In addition, the brain MRI results were normal. She had self-harmed since the first year of life, with scratching injuries to the face and bite injuries to the fingers. Neuropsychiatric evaluation defined a mild to moderate form of intellectual disability (verbal IQ 53, performance 59, total 49), abnormal behavior including a tendency to aggression toward both peers and adults, hyperactivity, obsessive-compulsive behavior, and non-compliance. She presented with autonomic symptoms in situations of high stress, such as anxiety, cold hands, sweating, mydriasis, and stiffening of the facial features, which led to real crises. The patient was repeatedly treated with antibiotics (mainly macrolides) for recurrent upper respiratory tract infections. Interestingly, during this period, both parents and doctors reported a general but significant improvement in behavioral aspects. In particular, the patient appeared less aggressive and less hyperactive, even after the acute period, when antibiotic therapy continued. Based on clinical features, genetic tests were conducted on her genomic DNA, including sequencing of a panel of genes for intellectual disability, array-CGH, and FRAXA. All tests were negative. After obtaining written informed consent from the parents, exome sequencing (ES) was performed on the proband and her parents gDNA.

### 4.3. ES

Exome sequencing was performed on the DNA samples of the proband and parent’s DNA samples using the Illumina Novaseq 6000 NGS platform (Illumina Inc., San Diego, CA, USA). Library preparation was performed using the BS Twist Human Core Exome (Twist Bioscience, South San Francisco, CA, USA), according to a specific protocol. Raw files (fastq) of each sample generated by the sequencing system were uploaded to the Sophia DDM platform v5.10.42 (Sophia Genetics, Rolle, Switzerland). The total amount of data already filtered (clean data), obtained by removing adapter contamination and low-quality sequences, was then mapped to the Human Reference Genome sequence (hg19) and then to the target regions. The analysis of these data is performed by the platform using both advanced bioinformatic tools and Sophia’s own patented software v5.10.42 that combine three specific algorithms for variant identification: (1) PEPPER for identification of SNVs and Indel; (2) MUSKAT for identification of CNVs; and (3) MOKA for variant annotation including different annotation tools (SIFT, Polyphen, MutationTaster, Mutation Assessor, FATHMM and GERP++ combined algorithm, LRT CADD, Varsome, Varsite and HOPE and Swiss Model; listed in section “web sources”). Variants were recalibrated using external datasets such as GnomAD (which includes 1000 Genomes and ExAC variants), ESP, COSMIC, and dbSNP (listed in section “web sources”). Variants were also filtered using custom-selected cutoffs to minimize false-positive detection. In particular, variants with allele frequencies < 2% and coverage ≥ 20× were retained. Variants that were reported to be benign or likely to be benign were excluded. The analysis of all remaining variants was initially conducted on trio data based on a “virtual gene panel” including potentially disease-causing genes correlated to the clinical signs presented by the patient. We then analyzed the data using a hereditary model approach by selecting all the de novo variants. The identified variants were validated using Sanger sequencing.

### 4.4. Sanger Sequencing

The c.196G>A (p.Gly66Arg) *AP1G1* variant was validated using standard Sanger sequencing. A 409 bp region was PCR-amplified using the primers *AP1G1-F* (5′-acatctctatttaatgtctttc-3′) and *AP1G1-R* (5′-ctggaaaggcatgatattgct-3′). Sequencing was performed on an ABI Prism 3500 genetic analyzer (PE Applied Biosystems Inc., Foster City, CA, USA), and the data were analyzed using Sequencher software V.4.9 (Gene Codes Corporation, Ann Arbor, MI, USA).

### 4.5. Mutation Nomenclature

All mutations were described according to the Human Genome Variation Society (HGVS) [41]. Nucleotide numbers were derived from the cDNA sequence of *AP1G1* (GenBank accession no. NM_001128).

### 4.6. Human Fibroblasts Cultures

Human fibroblasts from skin biopsies of the patient carrying the *AP1G1* variant and controls were obtained after obtaining informed consent and used at passage P6–10. A clinical description of the patients is reported in Section 4.2. Control fibroblasts were obtained from adults. Ethical approval for skin biopsies was obtained from the reference hospital centers.

### 4.7. Immunofluorescence Assay

For immunofluorescent studies, 4 × 104 AP1G1 mutant and control fibroblasts were seeded and cultured for 24 h or 48 h on glass coverslips. Cells were extensively washed with PBS, fixed at 20 °C with cold methanol for 7 min, permeabilized with 0.1% Triton X-100 in PBS at room temperature for 5 min and washed three times with PBS. After overnight incubation with blocking buffer (3% BSA, 1% glycine in PBS), the cells were subjected to immunolabeling using polyclonal anti TGN46, anti G130, anti AP1G1 (1:100; ABclonal, Düsseldorf, Germany) and goat anti-rabbit TRITC (1:50; Sigma Aldrich, Saint Louis, MO, USA) antibodies with fluorescein-conjugated goat anti-mouse IgG (Sigma-Aldrich) (1:50 in PBS, containing 3% BSA). After washing three times with PBS containing 0.1% Tween 20 and twice with PBS alone, the fibroblasts were incubated with 4′,6-diamidino-2-phenylindole (DAPI; Sigma Aldrich) (2 μg/mL) for 5 min and air-dried. The preparations were viewed under a DMRB Leica epimicroscope (Wetzlar, Germany) equipped with a digital camera.

### 4.8. In Vitro Synthesis of h WT AP1G1 and MUT AP1G1 mutantmRNAs

A total of 2 µg of recombinant plasmid pCS2+ containing the coding region of human *AP1G1*, both WT and mutant forms (Gly66Arg), was digested with EcoRI. The insert containing the specific *AP1G1* cDNA was gel-purified and transcribed with SP6 RNA polymerase using the MEGAscript SP6 in vitro transcription kit (Thermo Fisher Scientific, Waltham, MA, USA) according to the manufacturer’s instructions.

### 4.9. Zebrafish Maintenance and Eggs Collection

Zebrafish (*Danio rerio*) embryos were collected from three different lines: AB wild-type line, transgenic line Tg (*neurod*:EGFP), and CRISPR/Cas9-generated *ap1g1−/−* Adult fish were maintained under standard laboratory conditions in a circulating water system (Techniplast ZebTec, Buguggiate, Varese, Italy) at 27+/−1 °C in a 14 h light and 10 h dark daily cycle. The housing system guaranteed fish water (0.1 g/L^−1^ of Instant Ocean Sea Salts, 0.1 g/L^−1^ of sodium bicarbonate, and 0.19g/L^−1^ of calcium sulfate) at constant pH and conductivity values; ammonia, nitrite, and nitrate were kept below detection limits. Adult male and female animals were mated overnight in a breeding box. The next morning, freshly spawned eggs were collected, washed with fresh fish water, and maintained at 28 °C in 10 cm diameter Petri dishes containing fresh fish water until the onset of different embryonic stages, depending on the different experiments. Embryo staging was performed as described by Kimmel et al. (1995) [42].

### 4.10. Microinjections

In vitro-synthesized mRNAs of either human WT or MUT *AP1G1* mRNAs were injected with the dye tracer phenol red in 1X Danieau buffer (pH 7.6) into 1- to 2-cell stage embryos, according to an established protocol while not injected embryos were used as controls (CTRL) [43]. After microinjection, the embryos were incubated in fish water at 28 °C. To determine the optimal mRNA amount for further experiments, a dose–response curve was established. The following doses of each mRNA were injected: 10, 50, 100, and 250 pg/embryo. Survival rates and morphology were evaluated at 48 and 144 hpf. Data were obtained from four different experiments, with *n* = 30 for each experimental point.

### 4.11. Whole-Mount In Situ Hybridization (Wish)

Whole-mount in situ hybridization (WISH) experiments were performed according to standard methods as previously described [31]. All experiments were performed with embryos injected with the established optimal dose (50 pg/embryo) of either human WT or MUT *AP1G1* mRNAs at 16 and 48 hpf. Digoxigenin (DIG)-labeled antisense RNA probes for *neurogenin1* (*ngn1*) and *neuronal differentiation 1* (*neurod1*) genes were synthesized by in vitro transcription of linearized cDNA clones with T7 and SP6 RNA polymerases using DIG Labeling Mix (Roche, Basel, Switzerland) as previously described. Briefly, injected embryos at different developmental stages (hpf) were fixed overnight in 4% (*v*/*v*) paraformaldehyde (Sigma-Aldrich) at 4 °C, dehydrated through an ascending methanol (Sigma-Aldrich) series, and stored at −20 °C. After permeabilization with 10 µg/mL proteinase K (Roche), embryos were hybridized overnight at 68 °C. The following day, repeated washes were performed at high stringent temperature with saline-sodium citrate buffer (SSC) 2×/phosphate-buffered saline (PBS) 1× and SSC 0·2×/PBS 1X, then embryos were incubated with anti-DIG antibody (1:10.000) conjugated with alkaline-phosphatase (Roche) overnight at 4 °C. Staining was performed with nitroblue tetrazolium (NBT)/5-bromo-4-chloro-3-indolyl-phosphate (BCIP) (Roche) alkaline-phosphatase substrates according to the manufacturer’s instructions. Embryos were mounted in agarose-coated dishes, and WISH images were acquired using an AxioZoom V16 microscope equipped with a Zeiss Axiocam 506 color digital camera and processed using Zen 3.5 (Blue Version) software from Zeiss (Oberkochen, Germany). WISH experiments were performed in triplicate for both probes, and 30 embryos were analyzed for each experimental condition.

### 4.12. Microscopy

Bright-field images of embryos (anesthetized with tricaine 0.16 mg/mL embedded in 0.8% low-melting agarose and mounted on a depression slide) were captured using a Zeiss Axio Zoom V16 microscope equipped with a Zeiss Axiocam 506 color digital camera and processed using Zen 3.5 (Blue Version) software from Zeiss (Oberkochen, Germany). For light-sheet fluorescence microscopy analysis, embryos were first anesthetized using tricaine (0.02% in fish water) and subsequently embedded in a low-melting agarose matrix (Top Vision Low Melting Point Agarose, Thermo Fisher Scientific) at 0.5% in fish water. Images were acquired using a Zeiss Light Sheet microscope V1 supported by ZenPro software (Zen3.5 Blue version) using a 488 nm laser and 505–545 nm filter. Images from the same experiment were acquired using the same laser intensity and exposure time to obtain comparable data. After acquisition, 3D images were generated and manipulated using Arivis Vision 4D (Zeiss, Oberkochen, Germany). Three-dimensional reconstructions of EGFP-positive cells were manipulated to obtain images comparable to each other in terms of fluorescence intensities. Three-dimensional reconstructions were exported as single snapshots using the same compression settings.

### 4.13. Statistical Analysis

All graphs were plotted and analyzed using GraphPad Prism version 8 (GraphPad Software). Statistical significance was determined using one-way ANOVA with Tukey’s multiple comparison test. In all cases, *p*-values less than 0.05 were considered statistically significant (* = *p* < 0.05; ** = *p* < 0.005; *** = *p* < 0.001; **** = *p* < 0.0001). All data are presented as the mean ± standard error of the mean (SEM). Data from the behavioral experiments were analyzed using an unpaired *t*-test.

### 4.14. Locomotor Behavior Assessment

The injected embryos were maintained in fish water and incubated at 28 °C until 144 hpf. The light–dark locomotion test was performed as previously described [44]. Briefly, for each treatment, 12 larvae at 144 hpf were collected in a 96-square well plate with one larva per well and a volume of 200 μL. The 96-square well plate was then placed in the observation chamber of the *Danio Vision* Noldus system holder (Noldus, Wageningen, The Netherlands) in an isolated noise-free room. The larvae were allowed to adapt for 30 min before video recording. The system was then set up to track movements by recording the distance traveled in 2 min intervals over a 2 h observation period during 6 cycles of alternating light and dark 10 min periods. Data were analyzed using Noldus *Ethovision* software (XT 13.0 Version). Movements were reported as the total distance (mm) traveled by the larvae and speed (mm/s), calculated under both light and dark stimuli. Each light–dark locomotion experiment was repeated three times for both injected embryos, with 12 embryos in each group and each experiment.

### 4.15. Touch-Evoked Test

Non-injected and injected embryos were grown until 96 hpf. At this stage, 20 embryos were injected with either WT or MUT *AP1G1* mRNA and each of the three experiments was subjected to a touch-evoked test. Briefly, embryos were placed in Petri dishes beneath which a motility wheel was positioned, consisting of two concentric circles of increasing diameter (20 mm and >20 mm). Each embryo was initially placed at the center of a Petri dish, and the tail was gently touched with a smooth pipette tip. The touch response was observed and categorized as follows: (1) embryos that did not move, (2) embryos that swam < 20 mm, and (3) embryos that crossed the inner circle and swam > 20 mm. The percentages of each category (non-injected and injected) were calculated.

## Figures and Tables

**Table 1:** Morphological changes were observed at the different developmental stages. Percentage values were derived from three different experiments, with *n* = 40 for each experimental point. Control embryos (CTRL) were not injected.

Phenotype	CTRL	h AP1G1 WT mRNA	h AP1G1 MUT mRNA	Stage
**Abnormal body shape**	1%	1%	45%	**24 hpf**
	2%	3%	47%	**72 hpf**
**Brain abnormalities**	1%	3%	43%	**24 hpf**
	3%	3%	45%	**48 hpf**
**Pericardial edema**	2%	2%	40%	**72 hpf**
	3%	3%	42%	**144 hpf**
**Tail abnormalities**	2%	1%	39%	**144 hpf**
**Notochord**	1%	1%	32%	**144 hpf**

## Data Availability

The original contributions presented in this study are included in the article/Supplementary Material. Further inquiries can be directed to the corresponding author.

## Supplementary Materials

The following supporting information can be downloaded at: https://www.mdpi.com/article/10.3390/ijms262110590/s1.
