# Supplementary figures and images for: Usmani–Riazuddin Syndrome: Functional Characterization of a Novel c.196G>A Variant in the AP1G1 Gene and Phenotypic Insights Using Zebrafish as a Vertebrate Model
Source: Int J Mol Sci. 2025 Oct 30;26(21):10590. doi: 10.3390/ijms262110590 (PMC12610428; doi:10.3390/ijms262110590)

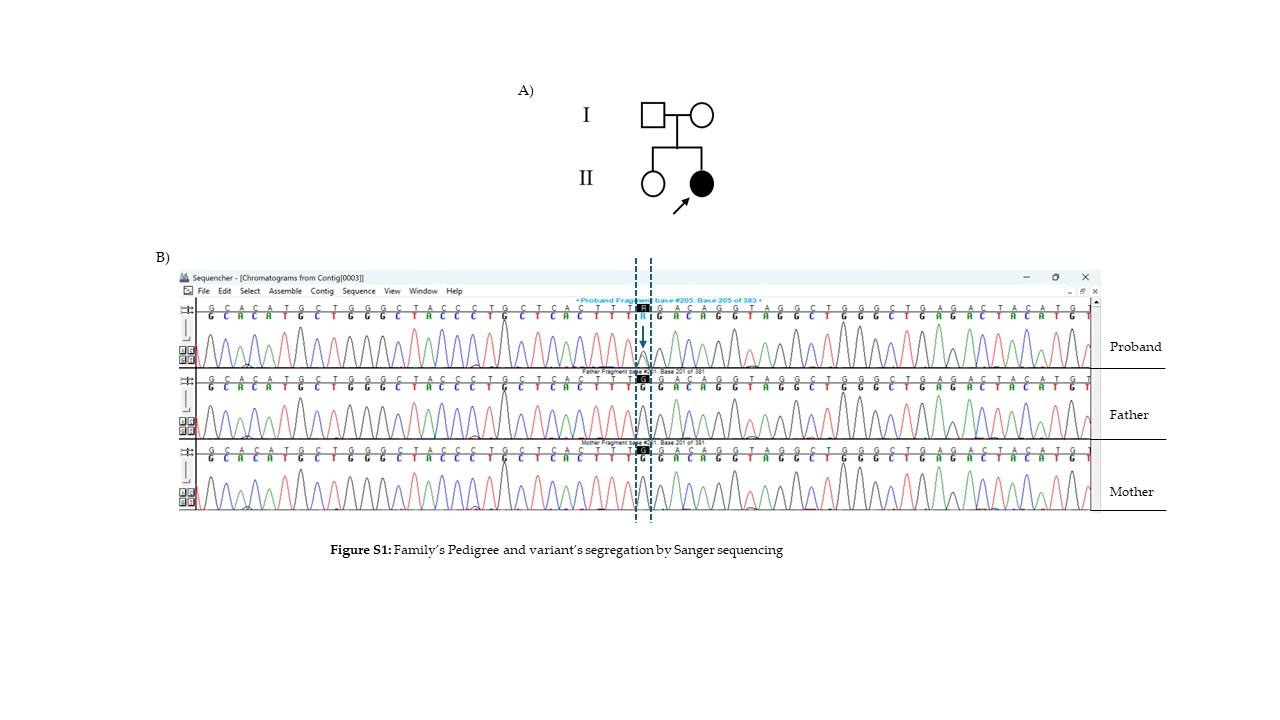

Supplement: Supplementary file 1 [file ijms-26-10590-s001.zip › Fig.S1_revised_Imperatore et al.tif]

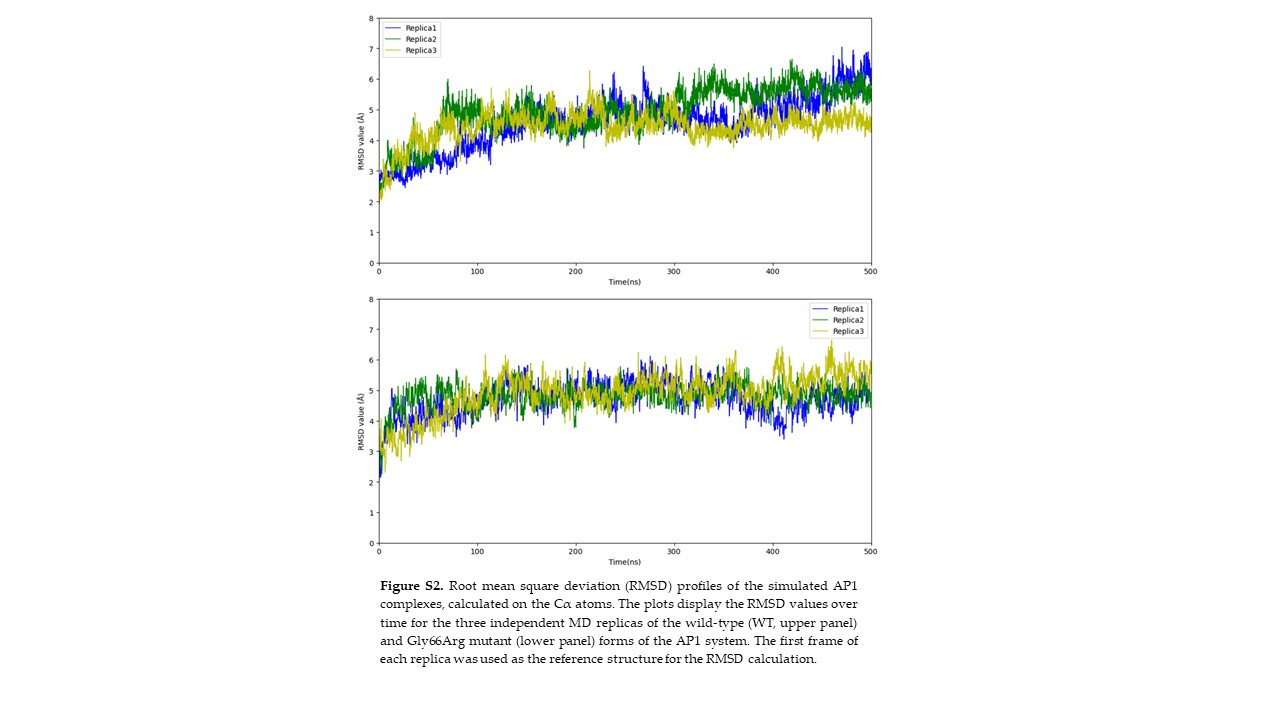

Supplement: Supplementary file 1 [file ijms-26-10590-s001.zip › Fig.S2_revised.tif]
